# A new method of predicting radial nerve location: a cadaveric study

**DOI:** 10.1007/s12565-025-00880-z

**Published:** 2025-07-30

**Authors:** Jianing Wang, Yadong Wei, Yudi Yang, Xiaodan Guo, Zhen Liu, Haiyan Lin, Xiangqun Yang

**Affiliations:** 1 Cadet Corps 6, Unit 16, Naval Medical University, Shanghai, China; 2 Department of Human Anatomy, Naval Medical University, 800, Xiangyin Road, Shanghai, China

**Keywords:** Humerus fracture, Radial nerve, Injury, Surgery, Olecranon

## Abstract

This study aimed to present a new method to predict an area with no Radial Nerve (RN) distribution (safe zone of the RN) in the middle and lower of the upper limb. The RN(60 in total) were dissected from 30 adult cadavers (20 males and 10 females). Acromial Angle, Olecranon, and Lateral Epicondyle of Humerus were selected as reference points. The linear projection of the RN on Acromial Angle–Olecranon and Acromial Angle–Lateral Epicondyle of Humerus were marked as ‘Intersection Point A’ and ‘Intersection Point B’. The lengths of Acromial Angle–Lateral Epicondyle of Humerus, Acromial Angle–Olecranon, Intersection Point A–Olecranon, Intersection Point B–Lateral Epicondyle of Humerus were measured. In this connection, the safe zone of the RN on the middle and lower Humerus was illustrated using Python3.11 and PyCharm2023.3.4. The mean position of Intersection Point A was located at (59.9 ± 6.2)% of the total length of Acromial Angle–Olecranon distally and the mean position of Intersection Point B was at (42.9 ± 6.6)% of the Acromial Angle–Lateral Epicondyle of Humerus. We identified absolute safe zone (with no RN distribution) on the middle and lower part of the Humerus enclosed by Olecranon, Lateral Epicondyle of Humerus, the 42.50% point of Acromial Angle–Olecranon distally, and the 28.10% point of Acromial Angle–Lateral Epicondyle of Humerus distally. In addition, relative safe zone (with less than 5% probability of the RN distribution) enclosed by Olecranon, Lateral Epicondyle of Humerus, the 50.33% point of Acromial Angle–Olecranon distally, the 32.98% point of Acromial Angle–Lateral Epicondyle of Humerus distally. Data in this study can help us locate the RN with body surface markers during Humerus fracture fixation.

**Electronic supplementary material:**

The online version of this article (10.1007/s12565-025-00880-z) contains supplementary material, which is available to authorized users.

## Introduction

Humeral fractures account for approximately 7–8% of all adult fractures in the western world (Court-Brown and Caesar 2006). The Radial Nerve (RN) originates from the posterior cord of Brachial Plexus, descends posterior to Axillary Artery, and passes through the radial groove to the anterior side of the arm near the junction of the middle and distal thirds of the humerus. In this connection, the RN is frequently involved in upper extremity fracture due to its long tortuous course and its close relation to the periosteum of Humerus (Mohler and Hanel 2006). In addition, surgical interventions for humeral fractures, such as plating, open reduction, or internal fixation, can cause the RN to be compressed or pulled. So a method for identifying the RN safe zone is essential to protect RN during surgery and to facilitate its localization in future revision surgeries.

Previous studies have established the anatomic relationship of the RN with various bony landmarks, such as medial epicondyle, Lateral Epicondyle of Humerus and the tip of the Acromion to aid surgeons in identifying the RN during surgical exploration of the Humerus (Bono et al. 2000; Carlan et al. 2007; Chou et al. 2008; Artico et al. 2009; Cox et al. 2010). In recent years, the point of confluence of the triceps aponeurosis, the point of deltoid insertion into the Humerus and fingerbreadths has been used as landmarks to identify, locate and protect RN intraoperatively (Patra et al. 2020; Yingling et al. 2020; Sapage et al. 2021). But the wide range of these proposed anatomic relationships limits the accuracy of the localized RN during the surgery.

In this study, 3 bony landmarks—Acromial Angle, Olecranon, Lateral Epicondyle of Humerus—were selected as fixed points. When measured, a white cotton thread was used to span between Acromial Angle and Olecranon, Acromial Angle and Lateral Epicondyle of Humerus. The linear projection of the RN with the line of the Acromial Angle–Olecranon and Acromial Angle–Lateral Epicondyle of Humerus were marked respectively as Intersection Point A and B. Data were obtained through measuring the distance of the Acromial Angle–Olecranon, Acromial Angle–Lateral Epicondyle of Humerus, the Intersection Point A–Olecranon, and the Intersection Point B–Lateral Epicondyle of Humerus. Distance of Intersection Point A–Olecranon was correlated to distance Acromial Angle–Olecranon to assess variability. So did distance of Intersection Point B–Lateral Epicondyle of Humerus to distance Acromial Angle–Lateral Epicondyle of Humerus.

## Materials and methods

The specimens used in this study comprised 60 sides of formalin–fixed upper limbs from 20 male and 10 female adult cadavers. Cadavers were obtained from body donation with informed consent, written and signed by the donator himself at body donation station of Navy Medical University. The dissection of cadaveric specimens following medical student dissection was performed according to routine rules at the department of human anatomy, Naval Medical University. The Institutional Review Board (IRB) at Naval Medical University indicated that IRB approval was not required for this study. The authors in this study hereby confirm that every effort was made to comply with all local and international ethical guidelines and laws concerning the use of human cadaveric donors in anatomic research.

**Fig. 1:**
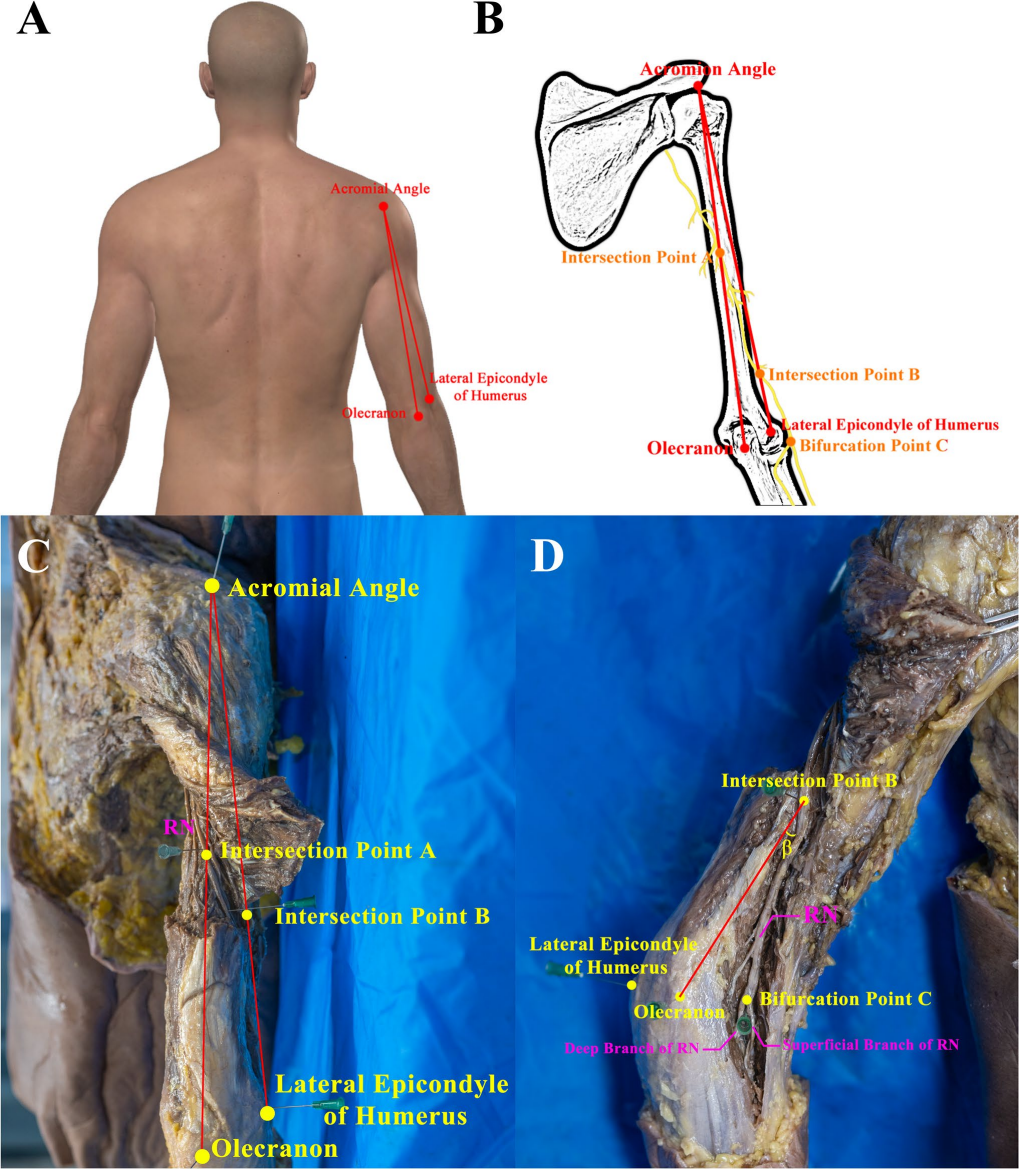
Photographs and schematic drawing of Acromial Angle, Olecranon, Lateral Epicondyle of Humerus, Intersection Point A and B to illustrate the measurement protocol at the posterior aspect of the right cadaveric upper limb. **A** Overview of the right cadaveric upper limb marked with the Acromial Angle, Olecranon and Lateral Epicondyle of Humerus. **B** Schematic diagram. **C** Exposed radial nerve and its vertical Intersection Point A and B with Acromial Angle–Olecranon and Acromial Angle–Lateral Epicondyle of Humerus separately. **D.** Illustration of ∠β and Bifurcation Point C

The cadaver was placed in a prone position with the upper arm abducted at a 30° angle to the chest wall. The Acromial Angle, Olecranon, and Lateral Epicondyle of Humerus were marked (Fig. 1A). Following medical student dissections of the upper limb, the lateral head of triceps brachii was exposed and transected, and the radial groove was exposed. The deep brachial artery and deep brachial vein are bluntly dissected, and the main trunk of the RN with its main branches was exposed in the middle segment of the upper arm bilaterally (Fig. 1C).

Intersection Point A is the linear projection of the RN on Acromial Angle–Olecranon. Intersection Point B is the linear projection of the RN on Acromial Angle–Lateral Epicondyle of Humerus (Fig. 1B, C). The bifurcation point of the RN into the superficial branch and deep branch was exposed and marked as Bifurcation Point C (Fig. 1D).

**Fig. 2:**
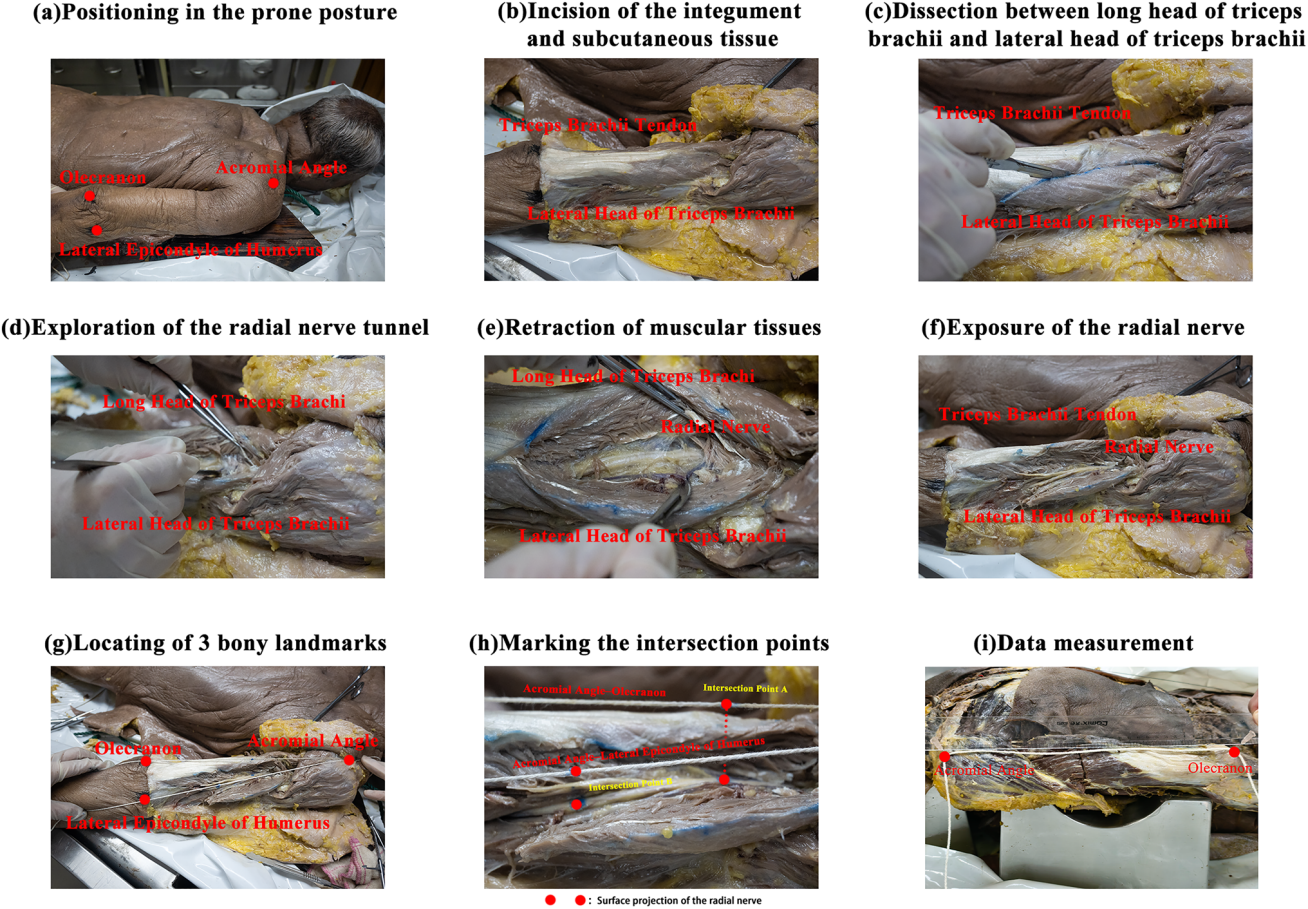
The process of disclosing the radial nerve (**a–f**). Confirmation of Olecranon, Acromial Angle and Lateral Epicondyle of Humerus with the help of the cotton line (**g**) as well as Intersection Point A and B (**h**) was shown. Points indicated by two needles stand for the position of Acromial Angle and Olecranon. Distance between two needles measured by a ruler is the distance of Acromial Angle–Olecranon (**i**)

Using a precision ruler with an error range of ± 0.5 mm, the distances of Intersection Point A–Olecranon, Acromial Angle–Olecranon, Intersection Point B–Lateral Epicondyle of Humerus, Acromial Angle–Lateral Epicondyle of Humerus, Intersection Point A–Intersection Point B and Intersection Point A–Bifurcation Point C were measured and recorded, respectively. The angle between Intersection Point B–Lateral Epicondyle of Humerus and Intersection Point B–Bifurcation Point C were measured using a precision protractor and denoted as ∠β (Fig. 1D). Distance of Intersection Point A–Olecranon/distance of Acromial Angle–Olecranon and distance of Intersection Point B–Lateral Epicondyle of Humerus/distance of Acromial Angle–Lateral Epicondyle of Humerus were calculated separately. To achieve consistent data, all the investigators had received the professional and technical training such as the localization of 3 bony landmarks and the criteria for data reading and recording. All measurements were taken three times, and the average values were recorded. The detailed procedure is attached in Fig. 2 facilitating others to repeat the experiment.

Data analysis was performed using IBM SPSS Statistics 26.0 and presented as mean ± standard deviation ($\overline{x}$±s). A *p* value less than 0.05 was accepted as statistically significant. All variables followed a normal distribution with Kolmogorov–Smirnov test.

In addition, distance of Acromial Angle–Olecranon and Acromial Angle–Lateral Epicondyle of Humerus from another 12 upper limbs was measured twice by the same investigator with the assistance of two other investigators. Then based on above data, 3 widely used precision estimates were calculated: the technical error of measurement (TEM), the relative technical error of measurement (rTEM) and the coefficient of reliability (*R*) (Goto and Mascie-Taylor 2007). TEM is the typical magnitude of error associated with precise measurement and can be used to estimate intraobserver precision. The TEM was calculated as the square root of the squared difference between 2 corresponding measurements divided by twice the sample size. rTEM represents an estimate of error magnitude as a percentage of object size. rTEM was calculated by dividing the TEM for a given variable by the mean for that variable and multiplying the result by 100. *R* represents the proportion of inter-subject variance free from measurement error and can be calculated using the following equation: *R* = 1 − [(TEM)^2^/(SD)^2^], where SD is the standard deviation of all measurements.

## Results

The distance of Acromial Angle–Olecranon and Acromial Angle–Lateral Epicondyle of Humerus from another 12 upper limbs (3 females’ and 3 males’) and TEM, rTEM and R for these variables were presented in Tables 1 and 2. TEM values were 0.05 to 0.08 mm and rTEM values were 0.32% to 0.56%. R values of all variables were 1, suggesting variation in the variables was due to factors other than measurement error. These data indicate an acceptable degree of intraobserver precision of the measurements.

**Table 1:** Distance of Acromial Angle–Olecranon and Acromial Angle–Lateral Epicondyle of Humerus measured twice (A and B) by the same investigator

Gender	Distance of Acromial Angle–Olecranon				Distance of Acromial Angle–Lateral Epicondyle of Humerus			
	Left		Right		Left		Right	
	A	B	A	B	A	B	A	B
Female	27.5	27.5	26.5	26.5	26.8	26.9	27.2	27.1
Female	29.2	29.4	30.0	30.0	28.6	28.6	28.7	28.8
Female	28.2	28.1	29.0	29.0	27.9	28.1	28.1	28.2
Male	30.0	30.1	30.1	30.1	29.0	29.1	29.6	29.6
Male	31.2	31.2	31.2	31.2	30.5	30.4	31.1	31.0
Male	28.9	29.0	29.5	29.5	28.7	28.8	29.0	29.1

**Table 2:** Precision estimates of measurements (*n* = 6)

Paraments	TEM	%TEM	*R*
Left Acromial Angle–Olecranon(mm)	0.05	0.37	1.00
Left Acromial Angle–Lateral Epicondyle of Humerus(mm)	0.06	0.40	1.00
Right Acromial Angle–Olecranon(mm)	0.08	0.56	1.00
Right Acromial Angle–Lateral Epicondyle of Humerus(mm)	0.05	0.32	1.00

Data from 20 male and 10 female adult cadavers were analyzed using Kolmogorov–Smirnov (K–S) test as shown in Table 3. Six items including the distance between Intersection Point A–Olecranon, distance of Intersection Point A–Olecranon/distance of Acromial Angle–Olecranon, distance of Acromial Angle–Lateral Epicondyle of Humerus, distance of Intersection Point B–Lateral Epicondyle of Humerus/distance of Acromial Angle–Lateral Epicondyle of Humerus, distance of Intersection Point A–Intersection Point B, and distance of Intersection Point B–Bifurcation Point C were normal distribution (*P* > 0.05),while three items including distance of Acromial Angle–Olecranon, distance of Intersection Point B–Lateral Epicondyle of Humerus and ∠β were not (*P* < 0.05). For these three items, their kurtosis absolute values were less than 10, and skewness absolute values are less than 3, indicating approximate normal distribution.

**Table 3:** Normal distribution analysis (*n* = 60)

Items	Mean	SD	Skewness	Kurtosis	Kolmogorov–Smirnov test	
					Statistic *D*	*P*-value
Distance of IPA-Ol	17.292	2.134	0.207	1.246	0.095	0.195
Distance of AA–Ol	28.852	1.961	-0.066	-0.508	0.118	0.038*
Distance of IPA–Ol/Distance of AA–Ol	0.599	0.062	-0.032	0.422	0.094	0.215
Distance of IPB–LEH	12.265	2.288	0.368	0.673	0.141	0.005*
Distance of AA–LEH	28.555	2.352	0.859	1.021	0.092	0.236
Distance of IPB–LEH/Distance of AA–LEH	0.429	0.066	0.025	0.260	0.090	0.260
Distance of IPA–IPB	4.785	1.656	0.458	-0.003	0.068	0.695
Distance of IPB–BPC	8.568	2.322	-0.257	-0.379	0.073	0.588
∠β	7.283	6.165	1.372	2.490	0.185	0.000*

*SD* standard deviation, *AA* acromial angle, *LEH* Lateral Epicondyle of Humerus, *IPB* Intersection Point B, *IPA* Intersection Point A, *Ol* Olecranon, *BPC* bifurcation point C

*P < 0.05

To evaluate the difference in data between males and females, the mean value of 9 items above were compared using *t*-test. As shown in Table 4, two relative items of distance between Intersection Point A–Olecranon/distance between Acromial Angle–Olecranon and distance between Intersection Point B–Lateral Epicondyle of Humerus/distance between Acromial Angle–Lateral Epicondyle of Humerus showed no difference between males and females, while absolute items such as distance between Acromial Angle–Olecranon and distance between Acromial Angle–Lateral Epicondyle of Humerus not (*P* < 0.05). In this connection, distance between Intersection Point A–Olecranon/distance between Acromial Angle–Olecranon and distance between Intersection Point B–Lateral Epicondyle of Humerus/distance between Acromial Angle–Lateral Epicondyle of Humerus were chosen as the two main referred items for locating the safe zone of the RN during posterior humerus surgery.

**Table 4:** Comparison of the distance, ∠β, IPA–Ol/AA–Ol and IPB–LEH/AA–LEH between males and females

Items	Gender (Mean ± SD)	
	Male (*n* = 40)	Female (*n* = 20)
IPA–Ol	17.60 ± 2.04	16.68 ± 2.23
AA–Ol	29.54 ± 1.77	27.48 ± 1.61*
IPA–Ol/AA–Ol	0.60 ± 0.06	0.61 ± 0.07
IPB–LEH	12.75 ± 2.20	11.30 ± 2.20*
AA–LEH	29.34 ± 2.33	26.98 ± 1.44*
IPB–LEH/AA–LEH	0.43 ± 0.06	0.42 ± 0.08
IPA–IPB	4.76 ± 1.62	4.84 ± 1.77
IPB–BPC	8.57 ± 2.40	8.57 ± 2.21
∠β	6.72 ± 5.29	8.40 ± 7.66

*SD* standard deviation, *AA* acromial angle, *LEH* Lateral Epicondyle of Humerus, *IPB* Intersection Point B, *IPA* Intersection Point A, *Ol* Olecranon, *BPC* bifurcation point C

*Compared to the male group, *p* < 0.05

The mean value of 9 items above between the right and left arm among all specimens was also compared. As a result, there was no significant difference as shown in Table 5 indicating the feasibility of choosing distance between Intersection Point A–Olecranon/distance between Acromial Angle–Olecranon and distance between Intersection Point B–Lateral Epicondyle of Humerus/distance between Acromial Angle–Lateral Epicondyle of Humerus as main referred items to localize the RN.

**Table 5:** Comparison of the distance, ∠β, IPA–Ol/AA–Ol and IPB–LEH/AA–LEH between the left and right arm

Item	Side (Mean ± SD)	
	Left (*n* = 30)	Right (*n* = 30)
IPA–Ol	17.05 ± 2.29	17.54 ± 1.98
AA–Ol	28.76 ± 1.94	28.94 ± 2.01
IPA–Ol/AA–Ol	0.59 ± 0.06	0.61 ± 0.06
IPB–LEH	11.87 ± 2.16	12.66 ± 2.38
AA–LEH	28.36 ± 2.15	28.75 ± 2.56
IPB–LEH/AA–LEH	0.42 ± 0.07	0.44 ± 0.06
IPA–IPB	4.76 ± 1.77	4.81 ± 1.57
IPB–BPC	8.60 ± 2.16	8.54 ± 2.51
∠β	6.62 ± 5.02	7.95 ± 7.15

*SD* standard deviation, *AA* acromial angle, *LEH* Lateral Epicondyle of Humerus, *IPB* Intersection Point B, *IPA* Intersection Point A, *Ol* Olecranon, *BPC* bifurcation point C

*Compared to the left, *p* < 0.05

**Fig. 3:**
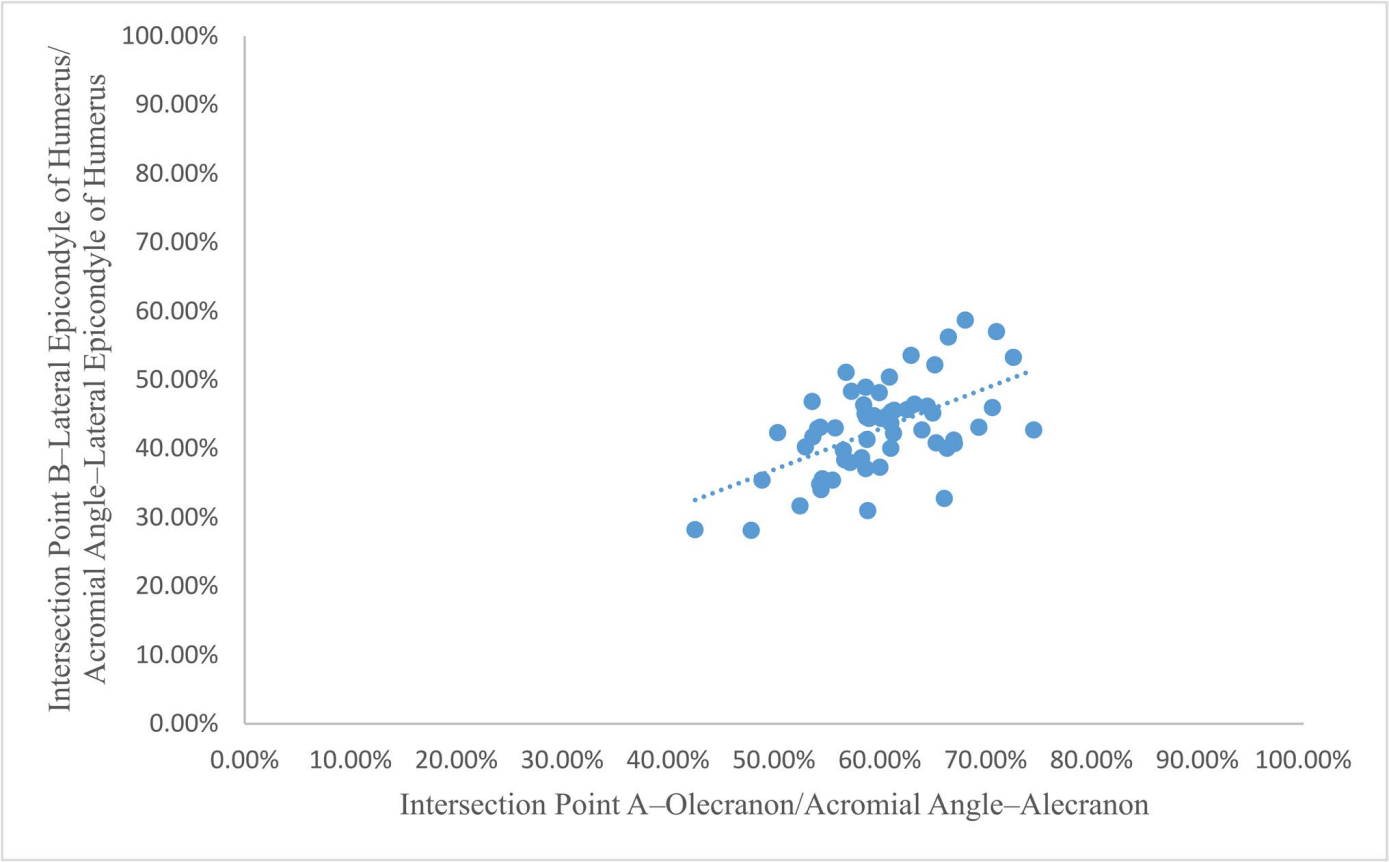
Regression analysis between distance of Intersection Point A–Olecranon/distance of Acromial Angle–Olecranon and distance between Intersection Point B–Lateral Epicondyle of Humerus/distance between Acromial Angle–Lateral Epicondyle of Humerus

In addition, distance of Intersection Point A–Olecranon/distance of Acromial Angle–Olecranon and distance of Intersection Point B–Lateral Epicondyle of Humerus/distance of Acromial Angle–Lateral Epicondyle of Humerus showed a positive correlation based on linear regression analysis and the regression coefficient of distance between Intersection Point A and Olecranon/distance between Acromial Angle and Olecranon is 0.593 (*t* = 5.107, *p* = 0.000 < 0.01, Fig. 3).

Based on 60 specimens, the mean value of distance between Acromial Angle–Lateral Epicondyle of Humerus was (28.555 + 2.352) cm and Acromial Angle–Olecranon (28.852 ± 1.961) cm respectively. The mean value of distance between Intersection Point A–Olecranon/distance between Acromial Angle–Olecranon and distance between Intersection Point B–Lateral Epicondyle of Humerus/distance between Acromial Angle–Lateral Epicondyle of Humerus was (0.599 ± 0.062) and (0.429 ± 0.066) respectively and the position of Intersection Point A and Intersection Point B was confirmed as a result (Table 3).

**Fig. 4:**
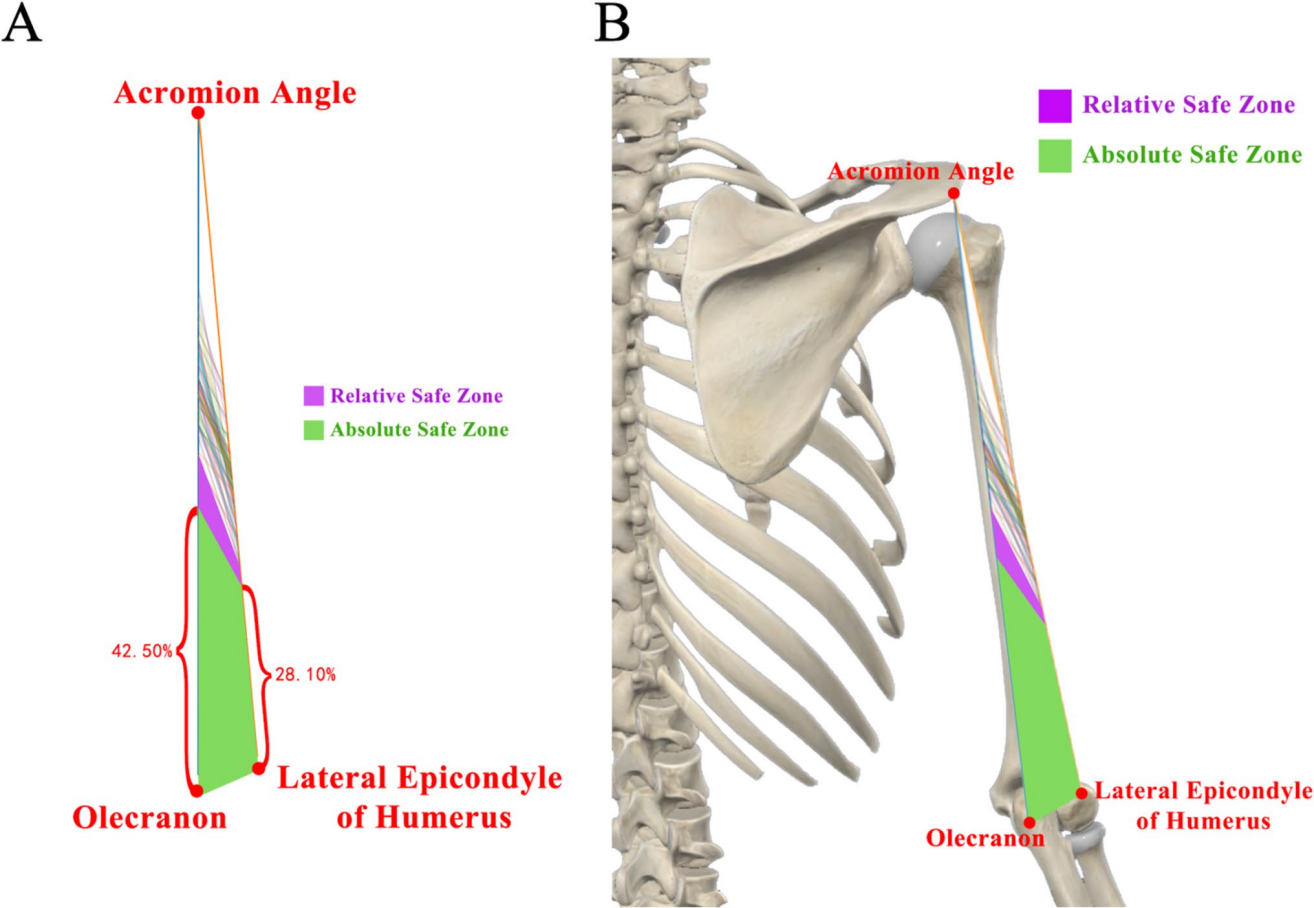
Schematic diagram of 60 RNs, absolute safe zone of the RN(green, zone with no RN distribution) and relative safe zone of the RN (purple, zone with less than 5% probability of RN distribution) at the posterior of the humerus

To visually illustrate the course of the RN in the middle and lower humerus, we used PyCharm 2023.3.4 to write a Python program for data visualization analysis. The specific steps are as follows: First, we constructed a triangular region based on 3 bony landmarks: the Acromial Angle, the Olecranon, and the Lateral Epicondyle of Humerus. Then, we called up the two main parameters, distance of Intersection Point A–Olecranon/distance of Acromial Angle–Olecranon and distance of Intersection Point B–Lateral Epicondyle of Humerus/distance of Acromial Angle–Lateral Epicondyle of Humerus, for all 60 sets of RN data. Using these parameters, our program drew all the lines AB, namely the RNs, within the triangular region and used different colors to distinguish each RN (Fig. 4A). Moreover, choosing proportional parameters can eliminate the bias caused by differences in humerus length among individuals.

We refined the zone with no RN or its branches distribution as absolute safe zone and with less than 5% probability of RN distribution as relative safe zone at the middle and lower part of the posterior humerus respectively. The absolute safe zone is surrounded by Olecranon, Lateral Epicondyle of Humerus, the 42.50% point of Acromial Angle–Olecranon length distally, and the 28.10% point of Acromial Angle–Lateral Epicondyle of Humerus length distally. The relative safe zone is surrounded by Olecranon, Lateral Epicondyle of Humerus, the 50.33% point of Acromial Angle–Olecranon length distally, the 32.98% point of Acromial Angle–Lateral Epicondyle of Humerus length distally (Fig. 4B).

**Fig. 5:**
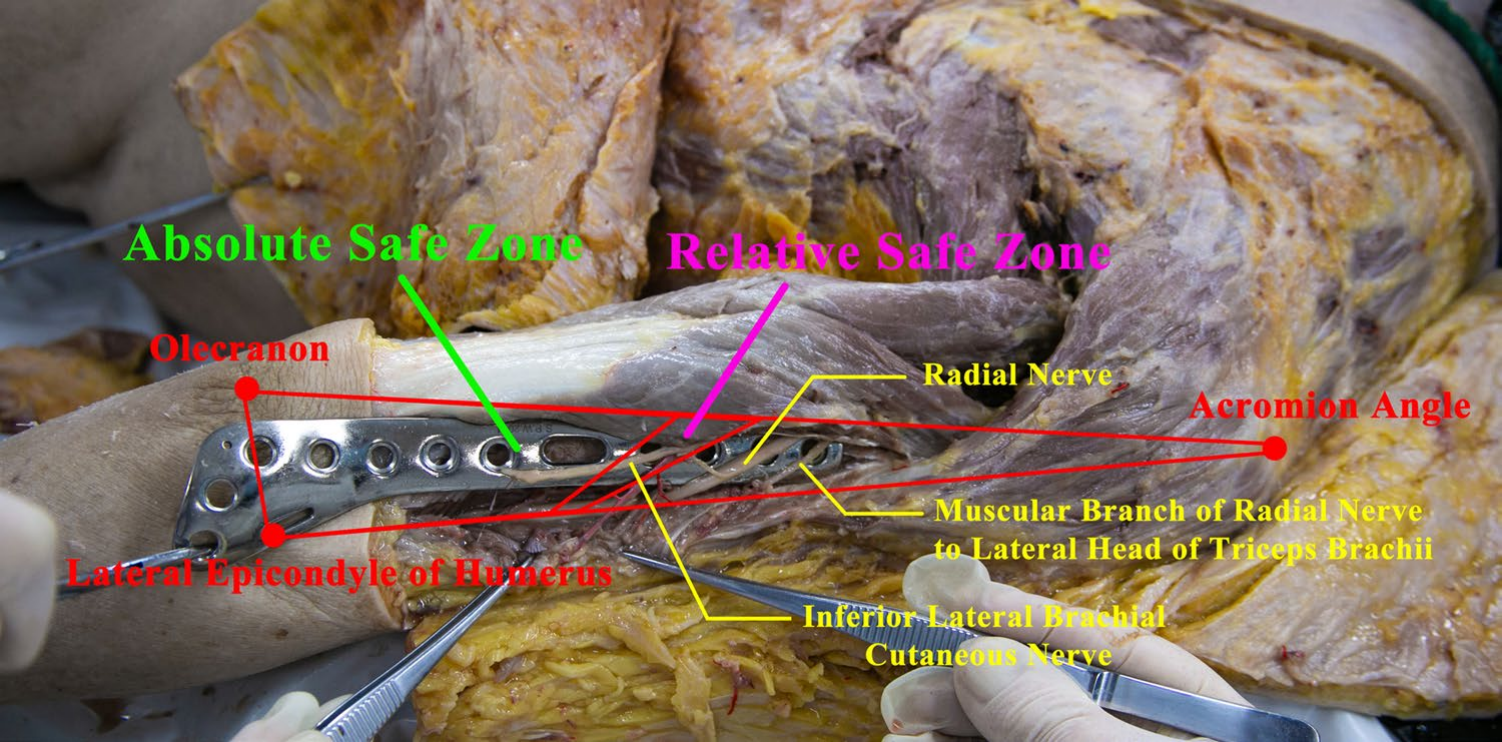
Illustration of the inserted plate, the radial nerve and its muscular branch to lateral head of triceps brachii and posterior brachial cutaneous nerve in the safe zone

In this study, the term “safe zone” does not suggest that there are no important blood vessels or RN branches within the muscle of the area. Rather, it specifically refers to the absence of the RN and its branches in the region adjacent to the humerus bone surface. This definition is crucial because iatrogenic injury to the RN during surgical procedures is predominantly caused by the insertion of plates near the bone surface, which can result in damage to the main trunk of the RN (Zhang et al. 2024). Consequently, when considering the safe zone, the blood vessels and nerves within the muscle tissue are not a primary concern. Illustration of the inserted plate, the radial nerve and its muscular branch to lateral head of triceps brachii and posterior brachial cutaneous nerve in the absolute safe zone also confirmed the safe zone we defined (Fig. 5).

**Fig. 6:**
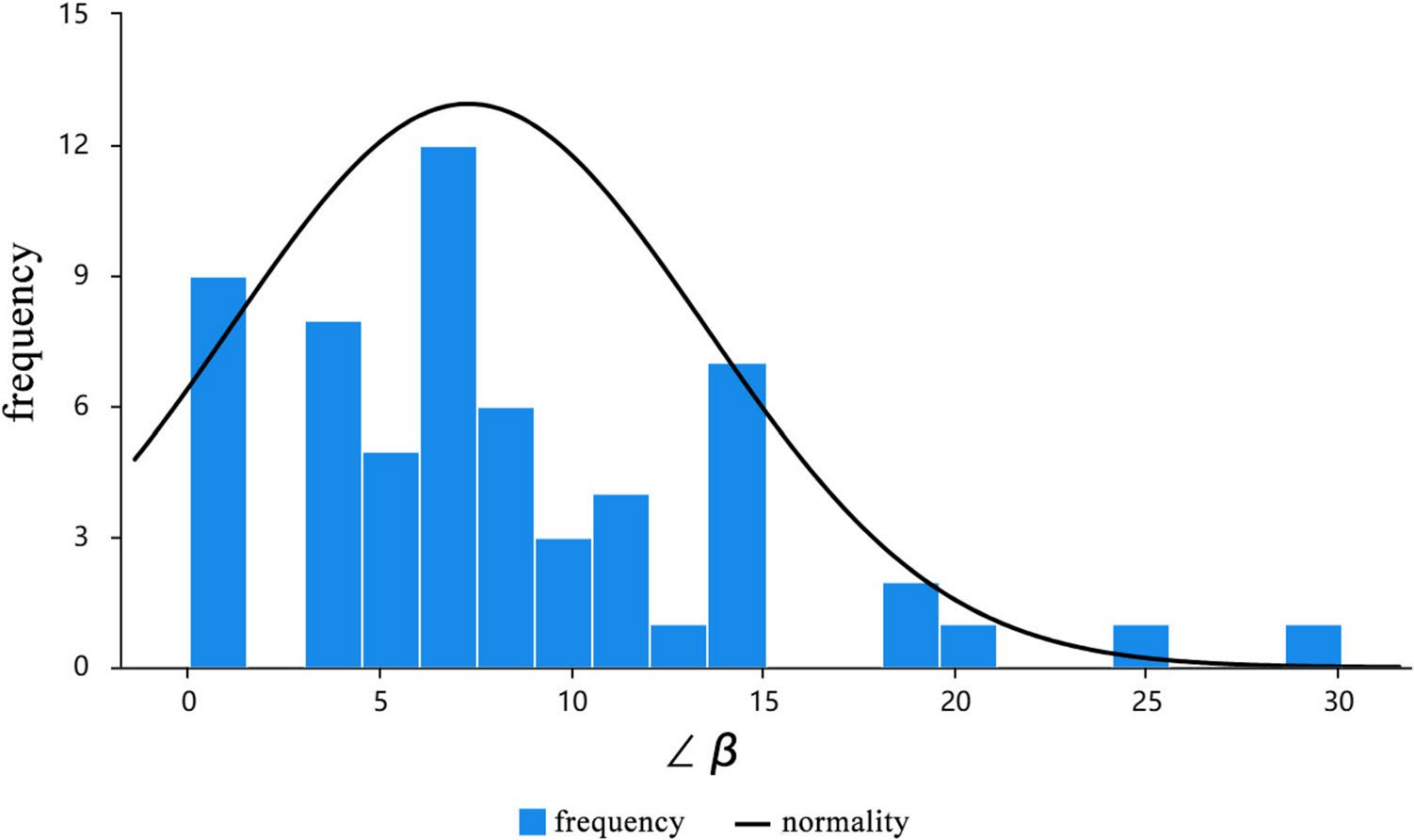
Frequency and normality of ∠β

The measurement of ∠β was primarily considered to serve the safe zone. However, it did not pass the normalization test (Fig. 6), indicating that the course of the RN becomes unpredictable after crossing the Acromial Angle–Lateral Epicondyle of Humerus. This is related to the gradual disappearance of muscle and connective tissues above this area, leading to increased mobility of the RN, which can even be palpated on the surface. Therefore, the frequency and normality of ∠β illustrates that the RN is highly variable outside the triangular region we defined.

## Discussion

Iatrogenic injuries to the RN commonly occur during the implantation of plates, due to compression or traction of the nerve. Inadequate exposure and lack of protection of the RN may be a significant cause of iatrogenic injury. In this study, by visualizing the data on the course of the RN in the middle and lower segments of the humerus, we found that there is considerable variation in the RN’s course within this region. This means that surgeons sometimes may need to perform extensive and time-consuming exploration to expose the RN. Due to the variation of the RN, we chose to define a safe zone rather than directly define the course of the RN, which was impossible. Surgeons can explore the RN outside the safe zone we provided, which can reduce the exploration area and improve efficiency. Our method can reduce surgical time, RN injury, and the occurrence of postoperative complications.

This study discussed the relationship between the RN and 3 bony landmarks, including Acromial Angle, Olecranon and Lateral Epicondyle of Humerus, to minimize the potential risk of surgical injury to the RN during surgeries on the middle and lower humerus.

Previous studies have reported using bony landmarks including deltoid tuberosity, Lateral Epicondyle of Humerus, Olecranon, and the proximal edge of the Olecranon fossa to localize the RN by the distance between RN and these bony landmarks (Carlan et al. 2007; Artico et al. 2009; Hackl et al. 2015a). Soft tissue landmarks, including the apex of the triceps aponeurosis, triceps tendon, and the confluence point between long and lateral heads of the triceps have also been discussed to identify the RN by measuring the distance to these landmarks (Arora et al. 2011; Seigerman et al. 2012; Patra et al. 2020). However, the distance between the RN and palpable landmarks usually varies with humeral length or triceps aponeurosis, decreasing the accuracy of RN localization during surgeries on the middle and lower humerus. Moreover, the bony landmarks can be used independently or combined with reported soft tissue landmarks to minimize the risk of the RN injury in humerus fracture fixation.

In this study, we chose Intersection Point A–Olecranon length/Acromial Angle–Olecranon length and Intersection Point B–Lateral Epicondyle of Humerus length/Acromial Angle–Lateral Epicondyle of Humerus length as main referred parameters to confirm the location of the RN, independent of individual difference such as humerus length and body height. In addition, Intersection Point A–Olecranon length/Acromial Angle–Olecranon length and Intersection Point B–Lateral Epicondyle of Humerus length/Acromial Angle–Lateral Epicondyle of Humerus length showed no gender-specific or side-specific difference and presented a strong positive correlation, indicating the RN consistently traverses the posterior of the Humerus from medial-superior to lateral-inferior without variations in other directions.

It has been published that the RN location on the Humerus varied based on the flexion Angle of the elbow joint, and a half-pin could be safely inserted, avoiding the elbow joint extension position (Hackl et al. 2015b; Sukegawa et al. 2018). However, the RN has diminutive shift between the obliquely oriented lateral intermuscular septum and the lateral aspect of the Humerus (Carlan et al. 2007).

Our data about ∠β further illustrate that the RN is highly variable outside the triangular region we defined. We illustrated all the 60 RN distribution at the posterior of the Humerus using Python3.11 and PyCharm2023.3.4 and calculated the absolute safe zone and relative safe zone to protect the RN during surgeries on the middle and lower Humerus.

This study is an anatomic investigation, and we did not have the opportunity to apply our findings in surgical procedures. By dissecting the relationship between the RN and the Humerus, we defined a “safe zone” as a region with the absence of the RN and its branches adjacent to the Humerus bone surface to provide an anatomic reference for clinical surgery. To minimize individual differences, the roles of the personnel were not changed during experimental process. Among our dissected 60 specimens, the vast majority of which were from elderly Chinese deceased individuals. Although it can’t meet with the variegated clinical conditions, methods in this study for calculating the RN safe zone can help the surgeons with its easy accessibility and universality.

In conclusion, this study provided the position of linear projection points of the RN on the line of Acromial Angle–Olecranon and the line of Acromial Angle–Lateral Epicondyle of Humerus to locate the RN safe zone with bony landmarks and reduce the RN injury during surgeries on the middle and lower Humerus.

## Electronic supplementary material

Below is the link to the electronic supplementary material.

Supplementary material 1 (XLSX 16 kb)

## Data Availability

All data supporting the findings of this study are available within the paper and the Supplementary materials.
